# Grip and Pinch Strength in Healthy Subjects and Patients with Primary Osteoarthritis of the Hand: A Reproducibility Study

**DOI:** 10.2174/1874325000802010086

**Published:** 2008-05-09

**Authors:** Efrat Ziv, Hagar Patish, Zeevi Dvir

**Affiliations:** 1 Department of Occupational Therapy, Sackler Faculty of Medicine, Tel Aviv University; 2 Department of Physical Therapy, Sackler Faculty of Medicine, Tel Aviv University; 3 Unit of Hand Surgery and Rehabilitation, Barzilai Medical Center, Ashkelon, Israel

**Keywords:** Hand, osteoarthritis, grip, pinch, strength, reproducibility.

## Abstract

Grip, Key Pinch (KP), 3 Point Pinch (3PP) and 2 Point Pinch (2PP) strengths were measured twice weekly in 32 women with primary osteoarthritis of the hand (POAH) and 25 healthy women. Reproducibility was assessed by standard error of measurement (SEM) and the coefficient of variation (CV). Cutoff values for significant improvement or deterioration were determined and expressed, respectively, as either the smallest detectable difference (SDD) or critical difference (CD). The SDD and CD of grip and pinch strengths were higher in POAH patients than in the healthy group. Among the pinch tests the 2PP findings were least reproducible. The relatively high SDD and CD scores indicate that improvement may be detected only in patients with moderate to severe weakness of grip and pinch. Furthermore, in POAH patients, diagnosing strength changes using the 2PP test is invalid due to low reproducibility.

## INTRODUCTION

Osteoarthritis (OA) is the most common joint disease in women aged 65 and beyond. It is characterized by weakness, pain and reduced functional ability [1, 2]. With respect to primary OA of the hand (POAH) the most affected joints are the distal interphalangeal, mainly of the index, the trapeziometacarpal joint, the proximal interphalangeal and the metacarpophalangeal [3].

In POAH patients undergoing treatment the aim of measuring grip and pinch strength is twofold: first to assess their hand strength status and second to monitor changes in these parameters during rehabilitation.

Reproducibility analysis is needed in order to decide whether the observed variations in the measurements indicate a true improvement or may be within the measurement error.

Reproducibility of hand strength measurements was reported in various studies [4-14]. Those measurements were largely based on the use of the so-called relative parameters such as Pearson's r or intra-class correlation coefficients (ICC). However, these parameters do not provide error margins. On the other hand, the use of absolute parameters such as the standard error of measurement (SEM) or the coefficient of variation of the standard deviation (CVp) lead to determination of cutoff scores which correspond to the amount of change that is beyond the natural individual variation of the criterion parameter. In order to set up such cutoffs at the individual subject or patient level, the SEM-based smallest detectable difference (SDD) [7, 8] or the CVp-based critical difference (CD) [9, 10] have been used.

The reproducibility of hand strength measurements in patients with POAH has not yet been reported. The main objective of this study was therefore to assess the reproducibility of hand strength measurements in patients with POAH in order to establish the cutoff values scores for a clinically significant change. To put the strength measurements as well as the resulting cutoffs in a more relevant perspective the measurements were also performed in healthy subjects of a similar age range.

## MATERIALS AND METHODOLOGY

### Subjects:

A convenience sample of 57 right hand dominant women, took part in the study. This group consisted of 32 patients (age range: 48-89 years; mean ± SD: 70.4 ± 10.0) suffering from POAH and 25 healthy subjects (age range 56-89 years; mean ± SD of 74.6 ± 8.4). A diagnosis of POAH was initially made by a general practitioner or the family physician and eventually confirmed by a hand surgeon according to the clinical criteria for the classification of primary hand OA [15]. The number of joints with clinical evidence of OA was recorded for both hands. Patients who had an upper extremity pathology which was unrelated to POAH were excluded. The healthy subjects were free of any hand pathology or typical OA of the hand as verified by a hand surgeon. This study was approved by the Human Experimentation Review Board of Tel-Aviv University.

### Instruments:

Grip strength was measured using Jamar grip dynamometer (Lafayette Instrument Co., Lafayette, Indiana). Pinch strength was measured using B&L pinch gauge (model pg-30; B&L Engineering, Santa Fe Springs, California). Pain was assessed using the visual analog scale (VAS) with no intermediate marking.

### Procedure:

Bilateral grip and pinch strength were measured twice (Test I and Test II) within one week (6.7 ± 1.6 days) by the same examiner. During the inter-testing interval the patients were not treated using conservative means but were free to take anti-pain medication. None of the patients started new medication. Grip measurement related to the second position of the dynamometer and was performed with the elbow at about 90° according to the American Society of Hand Therapists (ASHT) recommendations [16].

Pinch strength was measured with respect to the 3 standard positions: a. 2 point pinch (2PP, between the tip of the thumb and index finger) b. 3 point pinch (3PP, between the pad of the thumb and the pads of the index and middle fingers) and c. key pinch (KP, between the pad of the thumb and the medial-lateral surface of the index finger). Three consecutive measurements were performed with a 2 min inter-measurement interval [17].

### Data analysis:

Expressed in kgf (Kilogram Force) the criterion score for both grip and pinch strength was the average of the 3 repetitions. Paired t-test was used to compare the two trials and the numbers of OA involved joint in the right and the left hands. Test-retest Bland-Altman plots were used to examine the heteroscedasticity of the findings as well as the 95% limits of agreement [18]. The standard error of measurement (SEM) was calculated according to the following formula: SEM = SD*√ (1-r), in which SD is the standard deviation for both tests. The smallest detectable difference (SDD) was calculated as: 2.77* SEM. In addition, the pooled CVs (CVp) for group observations [10] for the two trials were calculated as follows:

$\mathrm{CVp}=100*\frac{\sqrt{\frac{\sum d^{2}}{2n}}}{\bar{x}}$

In which d is the difference in strength between the two trials and x is the mean strength from all the participants. The critical difference (CD, in %) was calculated as follows: CD = 2.77*CVp [9, 10].

## RESULTS

The average number of OA involved joints in the right hand (7.2) did not differ from that of the left hand (6.8, p=0.136). The mean VAS score among the patients was 4.5 ± 0.3 evidencing a moderate pain level. The mean and standard error of the grip and pinch strength for both groups are presented in Table **1**. No statistical differences were revealed between Test I and Test II except for a single instance: right hand grip strength in the POAH group (p=0.05). The strength findings for the two groups indicates that compared to their healthy counterparts POAH patients had on average a strength deficiency of 27% in grip and 24%, 32% and 28% in KP, 2PP and 3PP in pinch, respectively. The correlations (Pearson's r) between the VAS scores and strength were low and nonsignificant except for the case of 2PP (r=.34. p<0.05).

The Bland-Altman plots for 95% limits of agreement of the right hand in all strength measurements are presented in Fig. (**1**). The plots exhibit homoscedasticity namely the variance of the findings did not co-vary with the absolute strength. The same phenomenon was apparent in the left hand (not depicted pictorially).

For the determination of cutoff values, the SEM and CVp were used to derive the SDD and CD. These are presented in Table **2**.

## DISCUSSION

In the present study the reproducibility of grip and pinch strength scores was compared between healthy women and POAH patients. The findings indicate a relatively moderate hand muscles weakness in the patients supporting previous studies [19-21]. Since strengthening of hand muscles in patients with OA has been previously recommended [1, 22] monitoring change (improvement) in muscular capacity during the course of rehabilitation is integral to the process. Deciding whether observed differences are within the measurement error or signify a real improvement thus becomes a critical question which may be answered based on reproducibility analysis.

Prior to discussing the issue of reproducibility attention is drawn to the bilateral symmetry of severity. No significant difference was revealed with respect to the average number of clinically involved joints between the right and the left hand. These results are in agreement with some previous reports suggesting that in POAH both hands are involved [3, 23, 24] but are in variance with other which have argued that the severity of the disease was higher in the dominant hand [25, 26].

This is probably the first study which specifies cutoff scores for hand muscle strength in POAH patients. It is also distinguished by the fact that both parameters of reproducibility, the SEM and CVp, have been explored. We have chosen this double approach due to the increasing use of the SEM on the one hand and the expression of the CVp, on the other. Significantly, using the Bland-Altman plots, the findings reflected a homoscedastic distribution, which in terms of the SEM, may not allow transformation into relative (%) form. In other words, the measurement error remains stable irrespective of the patient’s baseline. Using the CVp does allow such view which quite surprisingly would have been in excellent agreement with the corresponding SDD scores if the SEM was divided by the mean value and multiplied by 100. It should be noted that application of the SDD (kgf units) discriminates somewhat against weak patients requiring relatively higher increase in strength. The opposite will be true for stronger patients when the CD is applied.

At any rate the results indicate that the detection of a real improvement in grip among POAH patients necessitates an increase of approximately 4.2 and 5.5 kgf in the right and left hands, respectively, upon application of the SDD. These values would respectively require an increase of 23% (right) and 36% (left) if one applies the CD. The difference between these cutoffs is due to a lower variability observed with respect to the right side which may reflect dominance and therefore better motor control. For pinch the SDD-based cutoff values were approximately 1 kgf for KP and 3PP whereas for the 2PP the values were larger, particularly relative to the baseline score. In terms of the CD an increase of approximately 30% signified, irrespective of side, a real change in KP and 3PP, while a much higher cutoff - 50% - was indicated for 2PP. We attribute the low reproducibility of the 2PP to the specific fingers alignment of this test position. In 2PP the tip of the index presses on the tip of the thumb. Since the distal interphalangeal joints are affected most frequently in hand OA [15, 3, 23] it stands to reason that pain which plays a major role in 'destabilizing' the measurements is behind the relatively large CD or SDD.

To put these findings in an appropriate perspective the cutoff scores obtained in previous reproducibility studies of hand muscles strength were analyzed. Schreuders *et al*. [12] calculated the SEM and resulting SDD in relation to the standard deviation of hand strength measurements in patients with a variety of hand injuries (average of 9 month after injury). Based on values given in their study we estimate the SDD to be 24% of the mean strength, a figure that is highly compatible with ours. In contrast, in a reproducibility study relating to grip strength in patients with rheumatoid arthritis and healthy subjects [14] the mean ± SD differences, assessed over a week interval, were 9.3 ± 9.4% and 10.1 ± 7.1% in the patients (n=10) and healthy subjects (n=12), respectively. Although not directly comparable to this present study, it is worth noting that Nitschke *et al*. [11] who assessed the measurement error of pain free grip strength in healthy and disabled (nonspecific regional pain) women indicated relatively low values of measurement error and no difference between the groups. Smidt *et al*. [13] tested the inter-observer reproducibility on the same day by calculating the standard errors of the measurement (SEM) and associated smallest detectable differences (SDD) of grip strength in patients with lateral epicondylitis. The SDD was smaller than 10% of the total range of measurements. Although comparison between the above and the current results is considerably hampered by the difference in patient population and test procedures, the low reproducibility of 2PP strongly suggests that it should not be used as a clinical indicator for monitoring change in POAH patients.

Substantially lower CD and SDD scores were found in the healthy group. Patient's motivation and pain, the testing device, the protocol and the tester can affect the recorded variations in strength. However both the healthy group and the POAH group were tested by the same tester using the same devices and protocol. It is also our impression that all were motivated to exert their maximal capacity in the tests. A possible explanation for the differences between the groups may lie in pain inhibition. Pain was indicated to be the main mediator between hand OA and hand strength [27]. Increase in the CD has been also noted in strength measurements among patients with low back pain and hip fractures [9, 28] However, as the correlation coefficient between the VAS and strength scores were low and largely insignificant this issue requires further exploration.

The above arguments notwithstanding, one should bear in mind that the derived cutoffs represent a clinimetric property of the test [7]. Such properties are a reflection of the statistical power of the test and should not be confused with what clinicians may deem as an improvement. In other words, it is possible that while a patient may not exceed the cutoff, and hence be *statistically* judged as staying at the same performance level, the clinical impression may point out otherwise. This possible clinimetric-clinical conflict has been recently highlighted [8] and given its importance deserves a thorough investigation with respect to a large number of performance parameters. However as the likelihood of a variance between the clinimetric criterion and the clinical impression is lower with increasing weakness, this study suggests that a real improvement may be detectable only in patients impaired with moderate to severe weakness of grip and pinch.

## CONCLUSION

Given the relatively high SDDs and CDs an improvement may generally be detectable only in POAH patients impaired with moderate to severe weakness of grip and pinch. The findings also indicate that the 2 point pinch test may not be used to detect strength change in POAH patients due to its low reproducibility in this specific pathology.

## Figures and Tables

**Fig. (1):**
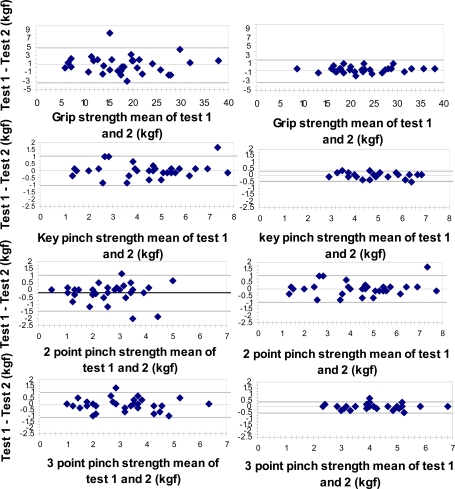
Bland-Altman plot of the test-retest differences of grip and pinch strength of the right hand in Primary Osteoarthritis of the hand (POAH) patients and healthy subjects. The horizontal (X) axis represents the mean value of Test1 - Test 2 in Kgf, vertical (Y) axis: The difference (Test 1 – Test 2) in Kgf. The central horizontal line represents the mean of the intra individual differences, and the dotted lines represent the 95% limits of agreement ( ± 2sd).

**Table 1:** The Test-Retest Mean ± SE of Grip and Pinch Strength (in kg Force) in with Primary Osteoarthritis of the Hand Patients (POAH) and Healthy Subjects

Healthy Subjects				POAH Patients				
Left Hand		Right Hand		Left Hand		Right Hand		
Test 2	Test 1	Test 2	Test 1	Test 2	Test 1	Test 2	Test 1	
17.7 ± 0.9	17.4 ± 0.8	20.8 ± 1.0	20.3 ± 1.0	15.0 ± 1.1	15.1 ± 1.2	17.8 ± 1.3	18.6 ± 1.3	Grip
4.7 ± 0.1	4.6 ± 0.1	4.9 ± 0.2	4.8 ± 0.2	4.1 ± 0.2	4.0 ± 0.2	4.2 ± 0.3	4.3 ± 0.2	KP
3.2 ± 0.1	3.2 ± 0.1	3.4 ± 0.1	3.4 ± 0.1	2.5 ± 0.1	2.3 ± 0.1	2.6 ± 0.2	2.4 ± 0.3	2PP
3.6 ± 0.1	3.6 ± 0.1	3.8 ± 0.1	3.8 ± 0.1	3.0 ± 0.2	2.9 ± 0.2	3.2 ± 0.2	3.1 ± 0.2	3PP

KP: key pinch, 2PP: two point pinch, 3PP: 3 point pinch.

**Table 2:** The Standard Error of Measurement (SEM), Smallest Detectable Difference (SDD), Coefficients of Variation (CVp) and Critical Difference (CD) in Primary Osteoarthritis of the Hand (POAH) Patients and Healthy Subjects

Healthy Subjects				POAH Patients				
SDD (kgf)	SEM (kgf)	CD (%)	CVp (%)	CD (%)	CVp (%)	SDD (kgf)	SEM (kgf)	
2.48	0.90	12.90	4.65	24.29	8.76	4.18	1.51	Griprt
0.40	0.14	8.42	3.03	23.24	8.39	1.00	0.36	Kp rt
0.54	0.20	16.11	5.81	51.35	18.59	1.27	0.46	2pp rt
0.47	0.17	12.14	4.38	32.13	11.59	1.02	0.37	3pp rt
1.94	0.70	11.52	4.15	36.24	13.08	5.47	1.98	Griplt
0.42	0.15	9.46	3.41	30.20	10.89	1.19	0.43	Kp lt
0.63	0.23	19.71	7.11	51.13	18.44	1.15	0.41	2pp lt
0.45	0.16	12.87	4.64	32.25	11.63	0.95	0.34	3pp lt

rt: right hand, lt: left hand, KP: key pinch, 2PP: two point pinch, 3PP: 3 point pinch.
